# Interferon regulatory factor 7 mediates obesity-associated MCP-1 transcription

**DOI:** 10.1371/journal.pone.0233390

**Published:** 2020-05-21

**Authors:** Masashi Kuroda, Misa Nishiguchi, Naho Ugawa, Etsuko Ishikawa, Yasuyo Kawabata, Saya Okamoto, Waka Sasaki, Yumiko Miyatake, Mayu Sebe, Saeko Masumoto, Rie Tsutsumi, Nagakatsu Harada, Hiroshi Sakaue

**Affiliations:** 1 Department of Nutrition and Metabolism, Institute of Biomedical Sciences, Tokushima University Graduate School, Tokushima-city, Tokushima, Japan; 2 Faculty of Food and Agricultural Sciences, Fukushima University, Fukushima-city, Fukushima, Japan; 3 Department of Health and Nutrition, Faculty of Nursing and Nutrition, The University of Shimane, Izumo-city, Shimane, Japan; 4 Diabetes Therapeutics and Research Center, Tokushima University, Tokushima-city, Tokushima, Japan; University of Texas Rio Grande Valley, UNITED STATES

## Abstract

Hypertrophy, associated with adipocyte dysfunction, causes increased pro-inflammatory adipokine, and abnormal glucose and lipid metabolism, leading to insulin resistance and obesity-related-health problems. By combining DNA microarray and genomic data analyses to predict DNA binding motifs, we identified the transcription factor Interferon Regulatory Factor 7 (IRF7) as a possible regulator of genes related to adipocyte hypertrophy. To investigate the role of IRF7 in adipocytes, we examined gene expression patterns in 3T3-L1 cells infected with a retrovirus carrying the IRF7 gene and found that enforced IRF7 expression induced the expression of monocyte chemoattractant protein-1 (MCP-1), a key initial adipokine in the chronic inflammation of obesity. CRISPR/Cas9 mediated-suppression of IRF7 significantly reduced MCP-1 mRNA. Luciferase assays, chromatin immunoprecipitation PCR analysis and gel shift assay showed that IRF7 transactivates the MCP-1 gene by binding to its proximal Interferon Stimulation Response Element (ISRE), a putative IRF7 binding motif. IRF7 knockout mice exhibited lower expression of MCP-1 in epidydimal white adipose tissue under high-fat feeding conditions, suggesting the transcription factor is physiologically important for inducing MCP-1. Taken together, our results suggest that IRF7 transactivates MCP-1 mRNA in adipocytes, and it may be involved in the adipose tissue inflammation associated with obesity.

## Introduction

Obesity is recognized as a prevalent global health problem. It is associated with increased risks of developing type 2 diabetes [1], cardiovascular diseases [2, 3], certain types of cancer [4] and depression [5]. Precise mechanisms are still unclear, but numerous studies demonstrated that adipocyte dysfunction and subsequent chronic inflammation are the primary defects linking obesity to whole body metabolism and cardiovascular diseases.

Energy imbalance can lead to adipose tissue expansion by an increase in adipocyte volume (hypertrophy) and number (hyperplasia). Between these, adipose hypertrophy is known to influence adipocyte biology and subsequently impair whole-body glucose homeostasis. Enlarged adipocytes exhibit functional disorders, characterized by impaired insulin sensitivity [6] and increased inflammatory adipokines, that induce immune cell activation and establish chronic inflammation [7]. Although adipocyte dysfunction may be secondary to hormonal changes resulting from excessive lipid accumulation, several studies have suggested that, at least to some degree, hypertrophy per se may be a cause of functional disorder in adipocytes. Of note, insulin sensitivity is inversely correlated with fat cell size, even after adjusting for body fat percentage [8, 9]. In addition, in a study with 108 healthy individuals followed for 9.3 ± 4.1 years, the authors found that enlarged fat cell size, and not high body fat percentage, was an independent predictor of diabetes and insulin resistance [8].

Functional disorders related to hypertrophic adipocytes are caused by transcriptional regulations at least to a certain extent [9]. Arner et al. evaluated the association between adipocyte morphology and gene expressions. Obese and non-obese subjects were divided into those with hyperplastic or hypertrophic adipocytes and the overall gene expression in adipose tissue was investigated. Principal component analysis clearly separated the four groups into three clusters: obese subjects (both with hyperplastic and hypertrophic adipocytes), non-obese subjects with hyperplastic adipocytes and non-obese subjects with hypertrophic adipocytes. In another study, it was demonstrated that adipocyte cell size was strongly associated with the transcriptome changes [10]. These findings indicated that hyptertrophy-associated functional disorder of adipocytes could be accompanied by the transcriptional changes, and that some kind of transcriptional regulators might be involved. In this study, our goal was to identify the transcription factors for obesity-associated gene expression and clarify their role.

We combined DNA microarray and motif analyses and identified IRF7 as a possible transcriptional regulator in enlarged obese adipocytes. We also found that this transcriptional factor can regulate the pro-inflammatory adipokine, MCP-1, and may contribute to triggering inflammation in adipose tissues of obese mice.

## Materials and methods

### Cell culture

3T3-L1 pre-adipocytes were kindly provided by Dr. Hosaka at the University of Shizuoka, Shizuoka, Japan. We maintained them at 37°C and 7.5% CO_2_/air in high-glucose Dulbecco’s Modified Eagle’s Medium (DMEM, Sigma, MO, USA) supplemented with 10% calf serum (Thermo Fisher Scientific, MA, USA). Confluent 3T3-L1 pre-adipocytes were kept for 48 hours and then induced into differentiation by replacing the media with DMEM containing 10% fetal bovine serum (GE Healthcare, Little Chalfont, UK), 10 μg/mL insulin (Wako, Tokyo, Japan), 500 μM isobutyl methylxanthine (Sigma, MO, USA), 1 μM dexamethasone (Wako, Tokyo, Japan), and 1 μM troglitazone (Cayman Chemical, MI, USA). After another 2 days, the media were replaced with 10% fetal bovine serum in DMEM and refreshed daily thereafter. Cells were harvested at days 2–8. For LPS stimulation, cultured adipocytes were starved without serum for 5 hours, and then incubated in antibiotic- and serum-free DMEM containing LPS at 10 ng/ml for 24 hours. For palmitic acid treatment, after 5 hours of serum starvation, 3T3-L1 adipocytes were exposed to 200 μM palmitic acid (Sigma, MO, USA) in 2% beef serum albumin (Sigma, MO, USA) / DMEM.

We maintained HEK293 cells and Plat-E cells at 37°C and 5.0% CO_2_/air in low-glucose DMEM (Sigma, MO, USA) supplemented with 10% fetal bovine serum. Cells were transfected with expression- and reporter-vectors, as described below.

### Retroviral vectors and infection

A retroviral vector encoding mouse IRF7 (IRF7-pMSCV) was kindly provided by Dr. Eguchi at the Okayama University Graduate School of Medicine, Okayama, Japan [11]. To generate retroviruses by transient transfection, we transfected Plat-E cells with pMSCV using Lipofectamine 2000 (Invitrogen, CA, USA). Two days after that, the supernatant containing retrovirus was collected. 3T3-L1 pre-adipocytes were incubated in retroviral media for 24 hours, and infected cells were selected by puromycin treatment.

### Transfection by electroporation

Differentiated 3T3-L1 adipocytes were electroporated using the NEPA 21 system (NEPA GENE, Chiba, Japan) according to the manufacturer’s protocol. Briefly, cells were trypsinized, washed in OptiMEM Reduced Serum Medium, suspended in OptiMEM Reduced Serum Medium at 50 × 10^4^ /100 μL, mixed with 100 μg expression vector, and transferred to a 0.2-mm gap cuvette. Then, 3T3-L1 adipocytes were plated in 24-well plates and cultured in 10% fetal bovine serum in DMEM for 24 h before use.

### Plasmid constructions

To generate reporter plasmids carrying mouse MCP-1 promoter, a 5′-flanking DNA sequence −2933 to −1055 nt, and −1157 to +71 nt (relative to the transcription start site) was amplified by PCR with PrimeSTAR® GXL DNA polymerase (TaKaRa, Tokyo, Japan). Gene-specific primers were as follows: 5′-aagctagcgaggatgaccagggaccaaa-3′ (*Nhe*I restriction site underlined) and 5′-ccggatccagcccttagaattcatttcagcag-3′ (*BamH*I restriction site underlined) for –2933 to –1055 nt (fragment A). 5′-ggaaatcaagatacctgagtggaag-3′ and 5′-ttggatccagagagctggcttcagtgagagtt-3′ (*BamH*I restriction site underlined) for −1157 to +71 nt (fragment B). Fragment A was digested with *Nhe*I and *BamH*I, and fragment B was with *BamH*I and *EcoR*I; they were inserted into the corresponding restriction sites of the pcDNA3.1(+) plasmid (Invitrogen, CA, USA). Each DNA fragment was confirmed by DNA sequencing. By combining fragments A and B, we obtained a 5′-flanking the DNA sequence of MCP-1 gene, and –2933 to +71 nt. This full-length MCP-1 promoter was excised using *Nhe*I and *BamH*I, and ligated into the pGL4.19 reporter plasmid (Promega, WI, USA). We introduced point mutations into the plasmid by PCR with GXL polymerase using primer pairs: 5′- cacagttAAtctcttccacttcctg-3′ and 5′-aagagaTTaactgtgggttggaatt-3′ for a mutation in ISRE –178 to –154 nt (uppercases indicate mutated bases). 5′- gcagctAAatttgctcccaggagtg-3′ and 5′- agcaaatTTagctgcgcagggagta-3′ for a mutation in ISRE −228 to −204 nt (uppercases indicate mutated bases).

To silence the IRF7 gene, we constructed the pSpCas9(BB)-2A-Puro (PX459) V2.0 CRISPR/Cas9 [12] against IRF7 exon 3. The target sequence was determined at crispr.mit.edu. Oligonucleotide pair (5′-caccgccactgcagcccctcgtac-3′, 5′-caccgccactgcagcccctcgtac-3′) was annealed and inserted into PX459 vector plasmid digested with the *Bbs*I restriction enzyme.

For the transient expression vector plasmid, we digested mouse IRF7 cDNA from the pCMV-IRF7 vector and subcloned it into the pcDNA3.1(+) or pcDNA3.1(+)/myc-HisA (Invitrogen, CA, USA).

### Establishment of IRF7 knocked-down cells

We transferred the PX459 against the IRF7 exon 3 into 3T3-L1 pre-adipocytes using the NEPA 21 system (NEPA GENE, Chiba, Japan) according to the manufacturer’s protocol. Transfected cells were selected by puromycin treatment (2.2 μg/ml) for 4 days. To increase genome editing efficacy, we repeated the transfections two times.

### Transfection and luciferase assay

HEK293 cells were plated in 24-well plates and transfected with reporter vector, IRF7 expression vector and β-galactosidase expression vector using Lipofectamine 2000. The following day, the cells were lysed with buffer supplied with the Luciferase Assay Kit (Promega, WI, USA). Luciferase activity was determined using a luminometer (Berthold Japan, Tokyo, Japan). Luminous intensity was normalized to β-galactosidase activity.

### 2-Deoxyglucose uptake measurement

3T3-L1 cells were seeded in a 6-well plate and induced into adipogenic cells according to the standard protocol. On day 7 after differentiation, mature 3T3-L1 adipocytes were serum-starved for 5 hours prior to measurements. Adipocytes were incubated with various concentrations of insulin in Krebs–Ringer Phosphate Hepes buffer (pH = 7.40) containing 2% beef serum albumin (Sigma, A8806) and then exposed to 1 mM 2-deoxyglucose (2-DG) for 20 min. The reaction was stopped by phlorizin treatment. 2-DG transported into the cells was measured using the Glucose Cellular Measurement Kit (Cosmo Bio, Tokyo, Japan) according manufacturer’s protocol.

### Oil red O staining

On day 7, 3T3-L1 adipocytes were fixed in 3.7% formaldehyde/PBS (−) for 16 hours at room temperature. Cells were then washed and stained with a filtered 0.35% Oil Red O solution in 60% isopropanol for 60 min at room temperature. After washing the cells in distilled water, they were observed under a BZ-X700 microscope (Keyence, Osaka, Japan).

### RNA isolation, reverse transcription, and quantitative real-time qPCR

We isolated total RNA from cultured cells or animal tissues using Trizol Reagent (Invitrogen, CA, USA) according to the manufacturer’s protocol. Complementary DNA was synthesized using TaKaRa PrimeScript RT reagent kits (TaKaRa, Tokyo, Japan), and analyzed by quantitative real-time PCR on a StepOnePlus^™^ (Applied Biosystems, CA, USA). We normalized gene expression to that of the 18S ribosomal RNA. Specific primer pairs are shown in Table 1.

**Table 1:** Primer pairs for real time qPCR.

Name of Genes	Forward primer (5’– 3’)	Reverse primer (5’– 3’)
mouse 18S ribosomal RNA	ggcctcgaaagagtcctgta	aaacggctaccacatccaag
mouse IRF1	tgtcgtcagcagcagtctctc	ttcggctatcttcccttcctc
mouse IRF2	ccttgcgggattgtattggt	tcagccactttagccctggt
mouse IRF3	ttgtgatggtcaaggttgttcc	tggaggtaggccttgtactggt
mouse IRF4	ttgaggaattggtcgagagga	gccatctgtgtgtcatccaaa
mouse IRF5	cacagagagccaacccactg	aggtggccacttggtgtctt
mouse IRF6	ccccatgactgacttggaca	tggacctgggaacttgacct
mouse IRF7	gagcgaagagagcgaagagg	ggcccacagtagatccaagc
mouse IRF8	tcaaggaaccttctgtggatga	ggagaaagctgaatggtgtgtg
mouse IRF9	aaccctaaccaaccac	gttgcagttgctgttgctgt
mouse MCP1	ctgttcacagttgccggctg	agcttctttgggacacctgct
mouse TNFα	caggcggtgcctatgtctc	cgatcaccccgaagttcaagtag
mouse Tap2	gcagacgacttcataggggaa	agttctgtagggcctgttcac
mouse IL1β	gccaccttttgacagtgatgaga	agctgccatccatccagaa
mouse IL6	cctctctgcaagagacttccatcca	agcctccgacttgtgaagtggt
mouse F4/80	ctttggctatgggcttccagtc	gcaaggaggacagagtttatcgtg
mouse leptin	agggaggaaaatgtgctgga	ggtgaagcccaggaatgaag
mouse adiponectin	ctacgaccagtatcagga	gaaagccagtaaatgtagag
mouse PPAR γ2	ccagagcatggtgccttcgct	cagcaaccattgggtcagctc
mouse C/EBP α	agcaacgagtaccgggtacg	tgtttggctttatctcggctc

### DNA microarray analysis

We purified total RNA with RNeasy mini kit (Qiagen, MD, USA) and synthesized cyanine-3 labeled cRNA using the One-Color Low Input Quick Amp Labeling kit (Agilent, Santa Clara, CA) according to the manufacturer's instructions. Labeled cRNA was hybridized with Agilent sureprint GE unrestricted microarrays (Agilent, Santa Clara, CA). Hybridized samples were scanned in an Agilent DNA microarray scanner (Agilent, Santa Clara, CA), and scanned images were analyzed using the Feature Extraction Software 10.7.1.1 (Agilent, Santa Clara, CA).

The results obtained were analyzed using GeneSpring software for characterizing upregulated genes during lipid accumulation. We submitted the list of upregulated genes to TransFind [13] to predict the genes’ transcriptional regulators.

### Subcellular fractionation and immunoblot

After treating the cells, as described in the Figure legends, we lysed and separated them into subcellular fractions using the Lysopure Nuclear and Cytoplasmic Extractor kit (Wako Tokyo, Japan) according to the manufacturer’s protocol. Samples were separated by electrophoresis onto a polyvinylidene difluoride (PVDF) membrane (Millipore, MA, USA) for transferring. The membrane was incubated in diluted primary antibody solution (1/500–1/1000) overnight (14–16 h) under agitation at 4°C. The PVDF membrane was incubated in horse radish peroxidase (HRP)-conjugated secondary antibody (1/10000 in TBST buffer) for 1 h. Signals were detected using a Clarity western ECL substrate (BioRad, Hercules, CA) and X-ray film (Carestream, NY, USA).

### Animal study and adipose tissue digestion

IRF7 knockout mice were provided by RIKEN BRC (Tsukuba, Japan) through the National Bio-resource Project of the MEXT, Japan. Starting when the mice were 4 weeks old, we fed them a control diet (Oriental Yeast, Tokyo, Japan) or a HFD60 (Oriental Yeast, Tokyo, Japan) containing 60% fat. All animal studies were conducted with male mice. Animal studies were performed according to guidelines of the Animal Research Committee of Tokushima University, and the Animal Research Committee of Tokushima University approved the protocols. The mice were humanely sacrificed by cervical dislocation.

We collected epidydimal white adipose tissue (eWAT), minced them, and digested them for 45 min at 37°C in PBS(−) supplemented with 2% bovine serum albumin (Sigma, MO, USA) and 2500 units/mL type II collagenase (Worthington, OH, USA). Digests were then incubated for 15 min in 10 mM EDTA, and passed through 250-μm nylon mesh. The filtrate was centrifuged at 1,200 rpm for 5 min at 4°C. We recovered adipocytes and stromal vascular fractions from the supernatant and pellet, respectively.

### Electrophoresis Mobility Shift Assay (EMSA)

HEK293 cells transfected with Empty- or IRF7-pcDNA were washed with PBS, and nuclear fraction was lysed and extracted in lysis buffer (20 mM HEPES-KOH(pH 7.9), 0.42 M NaCl, 1.5 mM MgCl_2_, 200 μM EDTA (pH 8.0) and 25% glycerol). Double strand DNA fragments for ISRE regions of mouse MCP-1 and their mutants (listed in Table 2) were labeled with ^32^P by T4 polynucleotide kinase reaction (TaKaRa, Tokyo, Japan) at 37°C for 30 min. Further, equal amount of extracts were incubated with 10 nM labeled DNA fragments and 10 μg/ml sonicated salmon sperm DNA in binding buffer (20 mM Tris-HCl (pH 7.5), 100 mM NaCl, 1.9 mM EDTA, 1.9 mM DTT, 10% Glycerol, 2% NP-40 and 0.6% beef serum albumin (Sigma, MO, USA)) at 24°C for 30 min. Samples were loaded on a 5% polyacrylamide gel and run in 0.5 TBE running buffer for 90 min at 150 V. The gel was dried and analyzed on a Fluorescent Imaging Analyzer FLA-9000 equipped with Multi-Gauge Version 3.0.

**Table 2:** DNA sequences for EMSA analysis.

Name of Probes	Forward (5’– 3’)	Reverse (5’– 3’)
ISRE (–228 to –204 nt)	cctgcgcagcttcatttgctcccaggag	ctcctgggagcaaatgaagctgcgcagg
Mutant for ISRE (–228 to –204 nt)	cctgcgcagctAAatttgctcccaggag	ctcctgggagcaaatTTagctgcgcagg
ISRE (–178 to –154 nt)	aacccacagtttctctcttccacttcct	aggaagtggaagagagaaactgtgggtt
Mutant for ISRE (–178 to –154 nt)	aacccacagttAAtctcttccacttcct	aggaagtggaagagaTTaactgtgggtt
Positive Control (PC)	gaaaactgaaagggagaaagtgaaagtg	tggaggtaggccttgtactggt

### Electroporation and Chromatin Immunoprecipitation (ChIP)- PCR

Empty- or myc tagged IRF7- pcDNA 3.1(+) was electroporated to matured 3T3-L1 adipocytes at day 5 using NEPA21 system (NEPA GENE, Chiba, Japan) according to the manufacturer’s protocol. Briefly, cells were trypsinized, washed and suspended in OptiMEM Reduced Serum Medium containing 100 μg plasmid at 50 × 10^4^ /100 μL. After electroporation, 3T3-L1 adipocytes were cultured in 10% fetal bovine serum in DMEM for 24 h before use.

Cells were trypsinized, crosslinked with 1% formaldehyde at 37°C for 15 min, and lysed with lysis buffer supplemented with PMSF and aprotinin. The lysate was sonicated to fragment genomic DNA, centrifuged, and supernatant was collected. Next, 1 μg of normal rabbit IgG (control) or anti-myc antibody and sepharose G beads were added to the resulting sample. After incubation with rotation for 4 hours, immunocomplex was precipitated with sepharose beads, and washed with cold buffer. The DNA/antigen/antibody complex was eluted with elution buffer and reacted with proteinase K at 45°C for 1 hour. Finally, DNA was purified with phenol/chloroform extraction and ethanol precipitation. The resulting ChIP DNA was analyzed real-time qPCR. The primer pairs are listed in Table 3.

**Table 3:** Primer pairs for ChIP PCR.

Name of primer pair	Forward (5’– 3’)	Reverse (5’– 3’)
–274 to –184 nt	ttactgccaattcttccctctttc	tttggtatttttctagccactcctg
–214 to –107 nt	gctcccaggagtggctagaa	tggaagttgaatccgctgag

### Measurement of adipocyte cell size

3T3-L1 adipocytes cultured for 8 and 20 days after the induction of adipogenesis, were detached, collected and fixed with osmium tetroxide for 3 days at 37°C. Cells were filtered with 10 μm nyron mesh, washed with PBS several times and collected in Isoton^TM^ II Dilutant (Beckman, CA, USA). Cell size was measured using Coulter Multisizer 3 (Beckman, CA, USA).

### Antibodies and other reagents

Anti-myc-Tag mouse mAb (Cell Signaling Technology, MA, USA #2276), anti-LaminA pAb (Santa Cruz Biotechnology, TX, USA sc-20680), anti-β-actin pAb (Proteintech, IL, USA 20536-1-AP), anti-p44/42 MAPK (ERK1/2) pAb (Cell Signaling Technology, #9102), anti-phosho-p44/42 MAPK (ERK1/2) (thr202/tyr204) pAb (Cell Signaling Technology, #9101), anti-Akt pAb (Cell Signaling Technology, #9272), and anti-phospho-Akt (ser473) pAb (Cell Signaling Technology, #9271) were diluted in TBST (1:1000) and used in immunoblot analysis. All other chemicals were analytical grade.

### Statistical analysis

We analyzed data using unpaired Student’s t-test and considered p-values of < 0.05 to indicate statistical significance.

## Results

### Identification of IRF7 as a possible transcription regulator in adipocytes

To identify the transcription factor associated with gene expression in large fat cells, we first conducted DNA microarray analysis of 3T3-L1 cells cultured for 2, 8 and 20 days after the induction of adipogenesis. We confirmed that as the culture period extended, lipid droplets apparently grew and the amount of cellular lipid accumulation increased (S1A–S1D Fig). Moreover, the mean adipocyte size at day 8 was 15.7 ± 0.11 μm whereas that at day 20 was 23.8 ± 0.33 μm (S1E and S1F Fig) suggesting that long-term culture leads to adipocyte hypertrophy. Real-time qPCR analysis demonstrated that these long-term cultured adipocytes reproduced the gene expression changes in adipose tissues in obese mouse (S1G–S1J Fig). Therefore, we thought that long-term cultured 3T3-L1 adipocytes were good *in vitro* model, which resembles large adipocyte in obesity.

**Fig 1:**
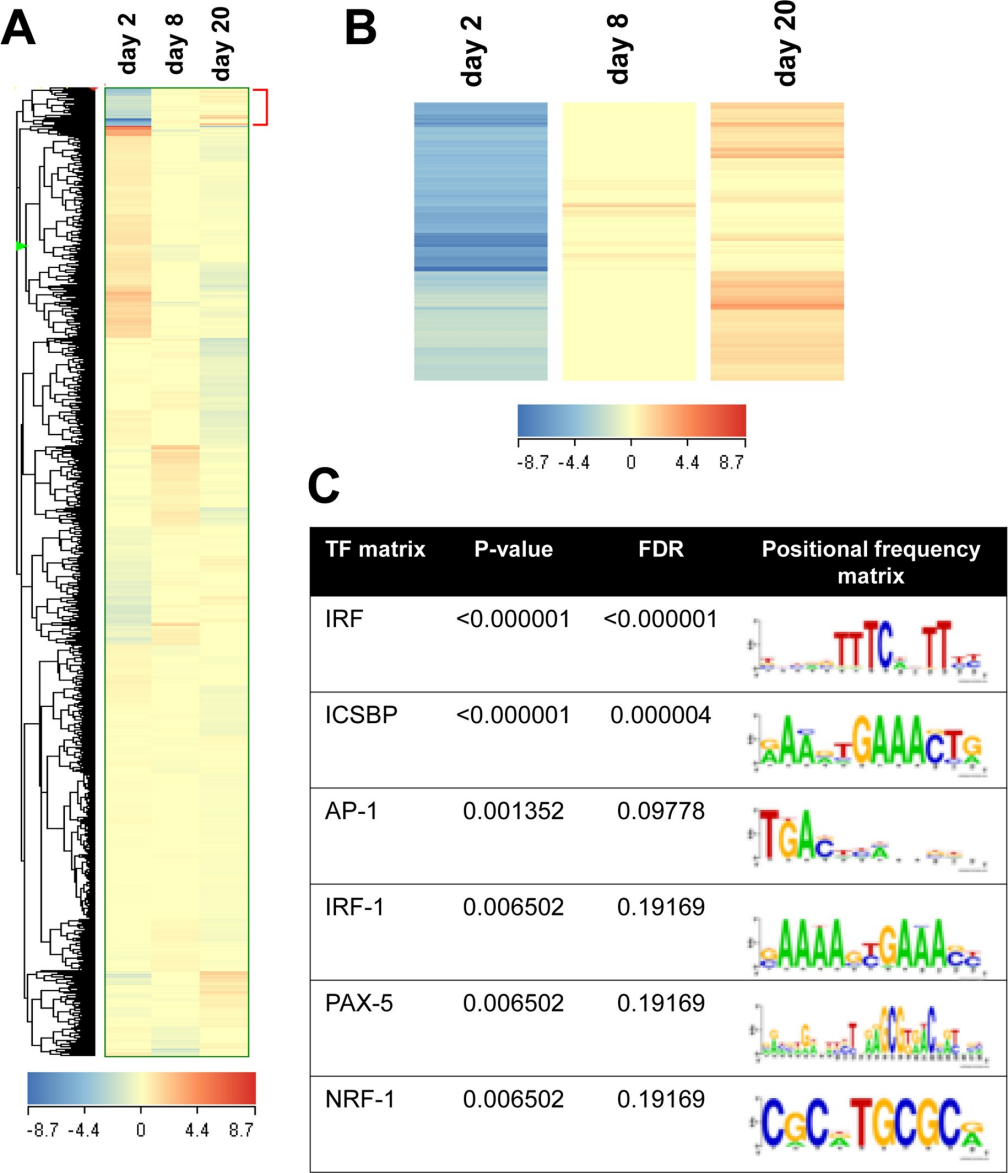
Identification of interferon regulatory factor 7. A and B: DNA microarray analysis was conducted in 3T3-L1 cells at 2, 8, and 20 days after inducing adipogenic differentiation. Hierarchical clustering analyses identified 503 genes that were upregulated in hypertrophied adipocytes. C: The identified gene set was submitted to TransFind, a software tool to predict the affinity of the transcription factor to promoter regions (−800 to +300 bp from transcription start site). A transcription matrix was defined as significant when the corresponding p-value was < 0.01. The output of the TransFind analysis was listed according to the p-value order. IRF: interferon regulatory factor, ICSBP: interferon consensus sequence-binding protein, AP-1: activator protein 1, PAX-5: paired box protein-5 and NRF-1: nuclear respiratory factor 1. FDR: false discovery rate.

**Fig 2:**
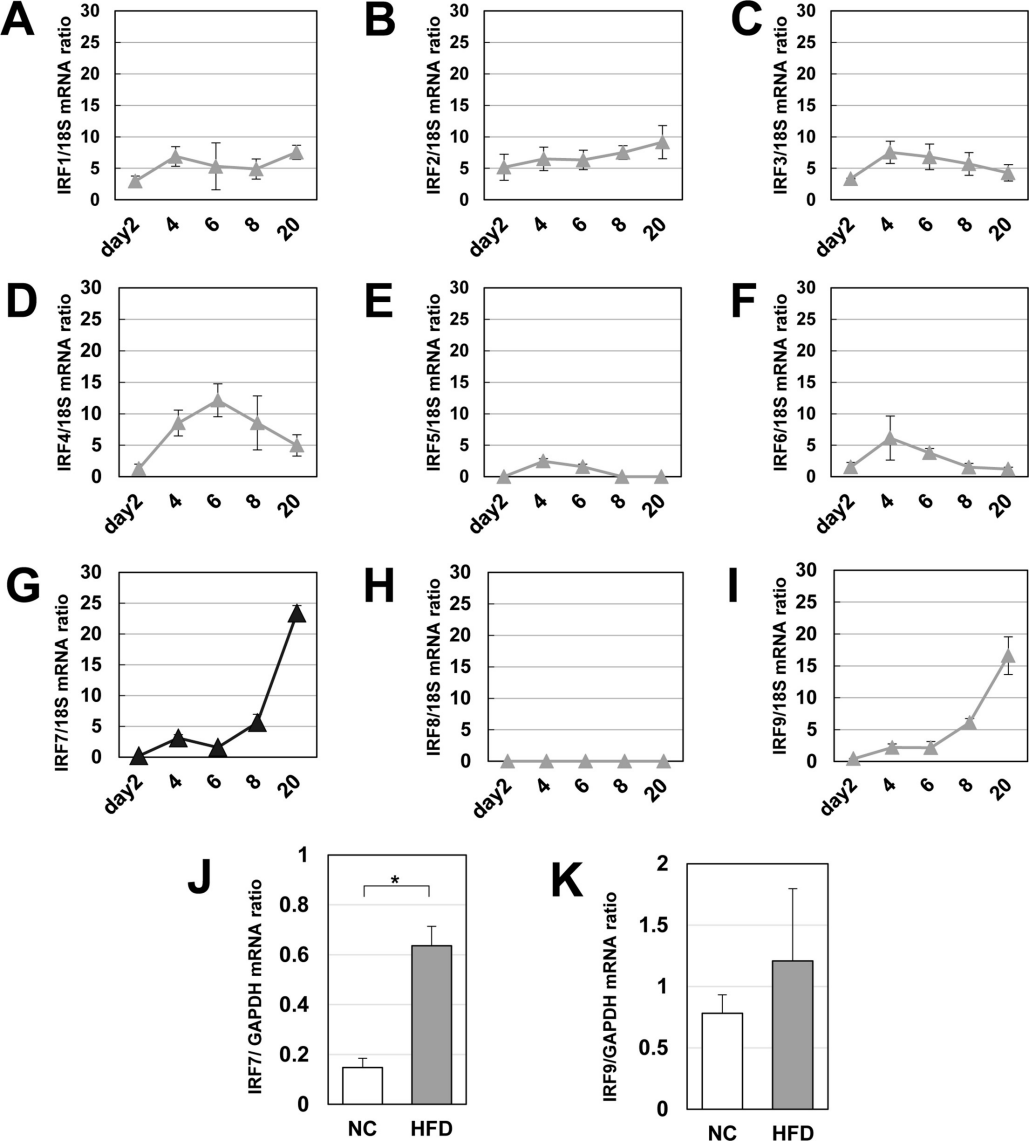
Expression pattern of IRFs in adipocytes during adipogenic differentiation and hypertrophy. A–I: The IRF1−9 mRNAs during adipogenesis and lipid accumulation in 3T3-L1 adipocytes (n = 4) were quantified by real-time qPCR. J and K: The mRNA levels of IRF7 and IRF9 in eWAT were determined by real-time qPCR. White adipose tissue was excised from C57BL/6J mice fed with normal chow (NC) or a high-fat diet (HFD) for 8 weeks starting at 4 weeks of age. *p < 0.05 and **p < 0.01. All values are means ± SEM.

We identified a set of 503 genes that increased from day 2 to day 20 (Fig 1A and 1B). Next, we performed a DNA sequence motif analysis of this gene set with TransFind [13] to predict possible transcriptional regulators in hypertrophied adipocytes. As listed in Fig 1C, we identified six transcription factor (TF) matrixes with p-values of < 0.01, and interferon regulatory factor (IRF) was listed at the top with the lowest p-value in six candidates. Among the IRF family members, IRF7 and IRF9 mRNAs increased with lipid accumulation, whereas other IRF members only slightly changed during early adipogenesis and lipid accumulation in adipocytes (Fig 2A–2I).

We also checked IRF7 and IRF9 mRNAs in white adipose tissues in obese mice. As shown in Fig 2J and 2K, IRF7 was robustly induced by a high-fat diet (HFD) (Fig 2J), whereas IRF9 tended to increase, but not significantly (Fig 2K). Thus, we selected the transcription factor IRF7 as our research target.

### Effects of IRF7 on MCP-1 mRNA expression

**Fig 3:**
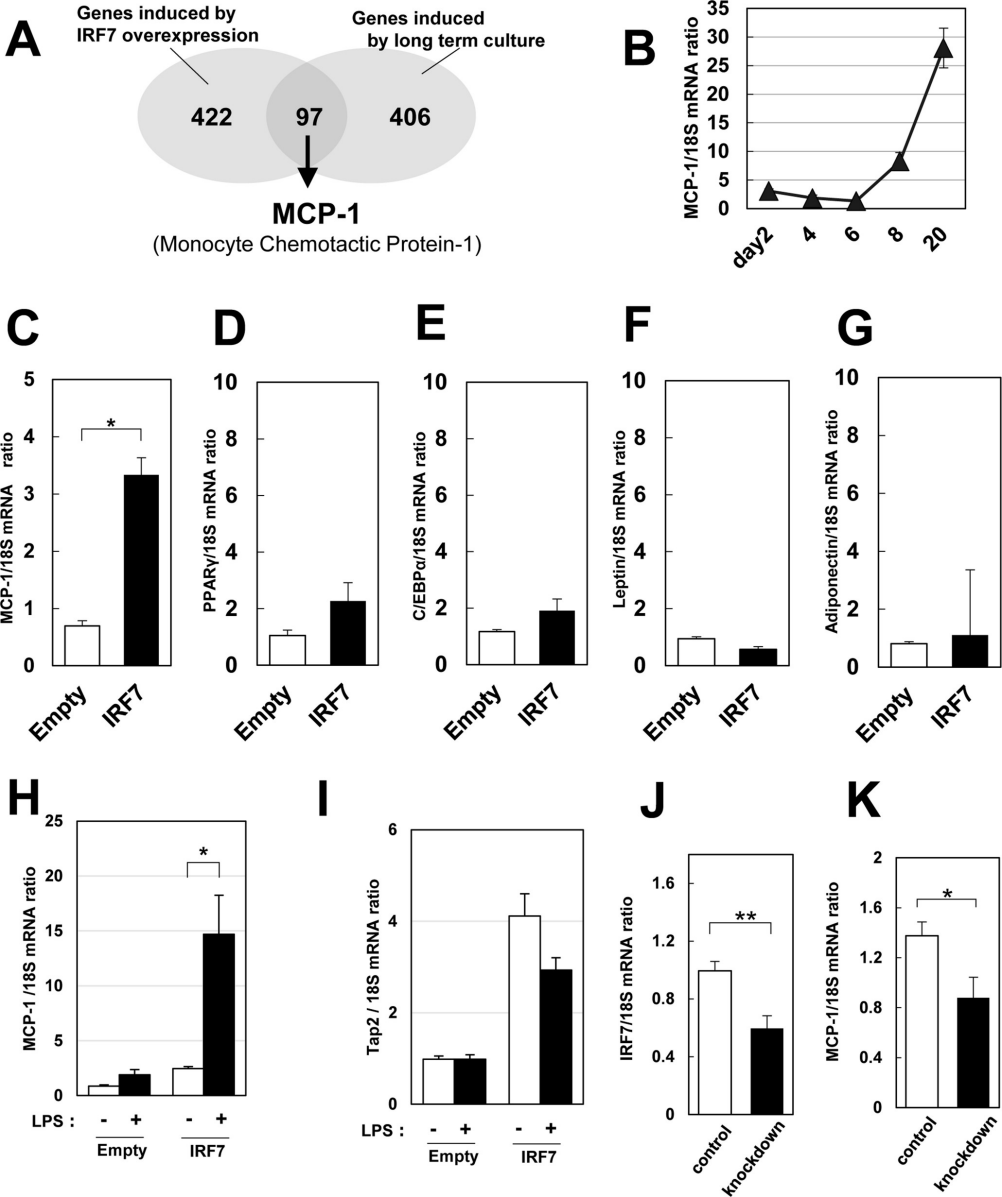
Identification of IRF7 target gene in adipocytes. A: The set of upregulated genes by IRF7 in 3T3-L1 adipocytes was obtained by DNA microarray analysis; 97 genes were overlapped with hypertrophy-induced genes. B: MCP-1 mRNA level changes during adipogenesis and lipid accumulation in 3T3-L1 adipocytes (n = 4). C–G: Empty- or IRF7-retrovirus infected 3T3-L1 pre-adipocytes were differentiated into adipocytes. 7 days after the induction of the differentiation, cells were harvested and analyzed by real-time qPCR (n = 4). H and I: Empty- or IRF7-retrovirus infected 3T3-L1 pre-adipocytes were differentiated into adipocytes. At day 6, cells were serum-starved for 5 hours and thereafter incubated in DMEM containing LPS at 10 ng/ml for 24 hours. Cells were harvested and analyzed by real-time qPCR (n = 4). J and K: IRF7 in 3T3-L1 cells were silenced by transfecting IRF7-PX459 into them and selected with puromycin. The resulting 3T3-L1 pre-adipocytes were differentiated into adipocytes. After 20 days of the induction into adipocytes, total RNA was isolated, and IRF7 and MCP-1 mRNA levels were examined (n = 5–6). *p < 0.05 and **p < 0.01. All values are means ± SEM.

To reveal the role of IRF7 in adipocytes, we first established IRF7-overexpressing 3T3-L1 cells by retroviral infection. No differences were observed between IRF7-overexpressing and control adipocytes in terms of the fundamental properties of adipocytes, including their morphologies, lipid accumulation, insulin receptor signal transduction, and insulin-induced 2-deoxyglucose transport (S2A–S2E Fig). To identify target genes of IRF7, we performed a DNA microarray again in 3T3-L1 adipocytes overexpressed with IRF7 and identified 519 genes that increased two folds or more. By comparing them with the 503 genes that were induced in adipocytes cultured for 20 days, we identified 97 genes whose expressions were elevated in both cases (by enforced induction of IRF7 and by long-term culture; see S1 Table). Among those genes, monocyte chemoattractant protein-1 (MCP-1) is the most studied chemokine and its involvement in obesity-associated inflammation is established well. Therefore, we focus more on MCP-1 (Fig 3A) whose expression pattern in adipocytes was very similar to that of IRF7 (Fig 3B).

Real-time qPCR analysis confirmed IRF7 over-expression induced MCP-1 mRNA, but did not affect other adipogenic genes, like PPAR γ, C/EBP α, leptin, or adiponectin (Fig 3C–3G). The effects of IRF7 on MCP-1 expression became more evident when cells were exposed to lipopolysaccharide (LPS) (Fig 3H). On the other hand, the typical IRF target gene, Tap2, was elevated in IRF7-overexpressed cells, but was not affected by LPS stimulation (Fig 3I), indicating the synergistic effects of IRF7 and LPS stimulation were specific to MCP-1 transcription.

Contrary to that, when IRF7 was depleted by CRISPR/Cas9 genome editing in 3T3-L1 adipocytes, the amount of MCP-1 mRNA decreased (Fig 3J and 3K).

### Effects of IRF7 expression on MCP-1 promoter activity

**Fig 4:**
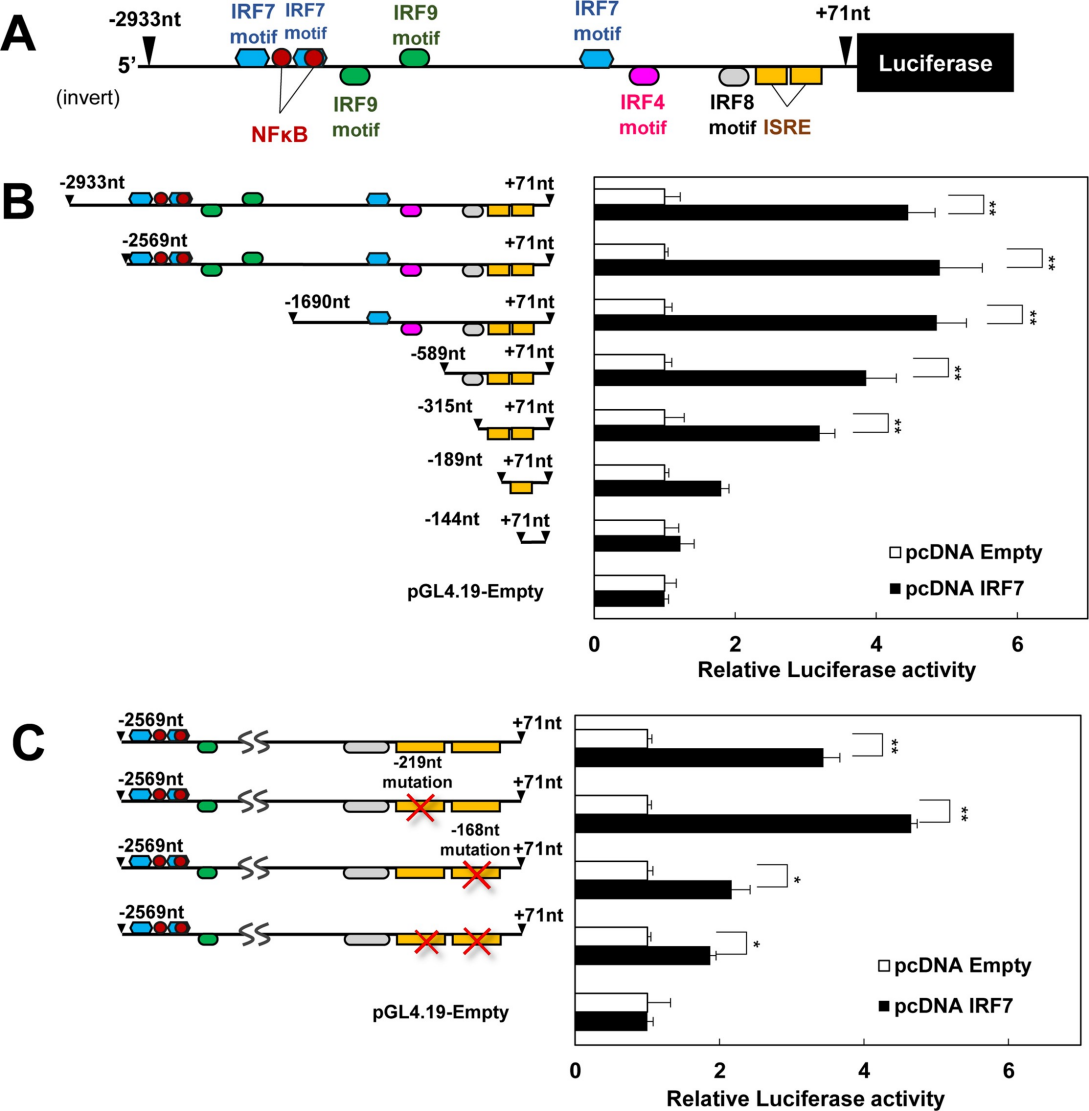
Effects of IRF7 expression on promoter activity of murine MCP-1. A: The promoter −2933nt to +71nt of the mouse MCP-1, upstream of the transcription start site, was inserted into the pGL4.19 reporter vector. Putative binding motifs are shown. B: The activity of MCP-1 promoter was measured in HEK293 cells with various lengths of 5’-fragments. Results are shown as fold inductions relative to the values in the cells transfected with Empty-pcDNA vector (n = 4). C: Luciferase assay performed in HEK 293 cells with MCP-1 promoter- pGL4.19 vector carrying one or two proximal ISRE mutations. Results are shown as fold inductions relative to the values in the cells transfected with Empty-pcDNA vector (n = 4). *p < 0.05 and **p < 0.01. All values are means ± SEM.

To confirm MCP-1 was induced by IRF7, we examined the effects of IRF7 over-expression on the mouse MCP-1 promoter activity. We isolated the mouse MCP-1 promoter ranging from –2933 to +71 nt, and inserted it into the pGL4.19 reporter vector (Fig 4A). As shown in Fig 4B, the MCP-1 promoter was markedly activated by IRF7 over-expression. Several sequence motifs might be bound by IRF7 within our examined promoter region (Fig 4A). To identify the sequence responsible for IRF7-mediated induction, we performed a promoter assay with a construct containing a series of 5′- deletion mutants (Fig 4B). Deleting the sequence from –2933 to –315 nt did not affect the MCP-1 promoter activation by IRF7, but further deletion attenuated the effect of IRF7 on the promoter activity, indicating the presence of relatively important motifs within the sequence of −315 nt or less. This region had putative IRF7 binding ISRE inverted motifs from −228 to −204 nt and from −178 to −154 nt, and we generated constructs containing different mutations in those ISREs (Fig 4C). The promoter construct with a −219 nt mutation was activated by IRF7 to the same extent as the wild type promoter. Although there was a significant induction of promoter activity of the reporter vector with a −168 nt mutation, the degree of activation was lower than that of the wild type promoter vector, implying that the ISRE ranging from −178 to −154 nt plays a relatively important role in MCP-1 transactivation by IRF7.

### Binding of IRF7 to MCP-1 promoter in adipocytes

**Fig 5:**
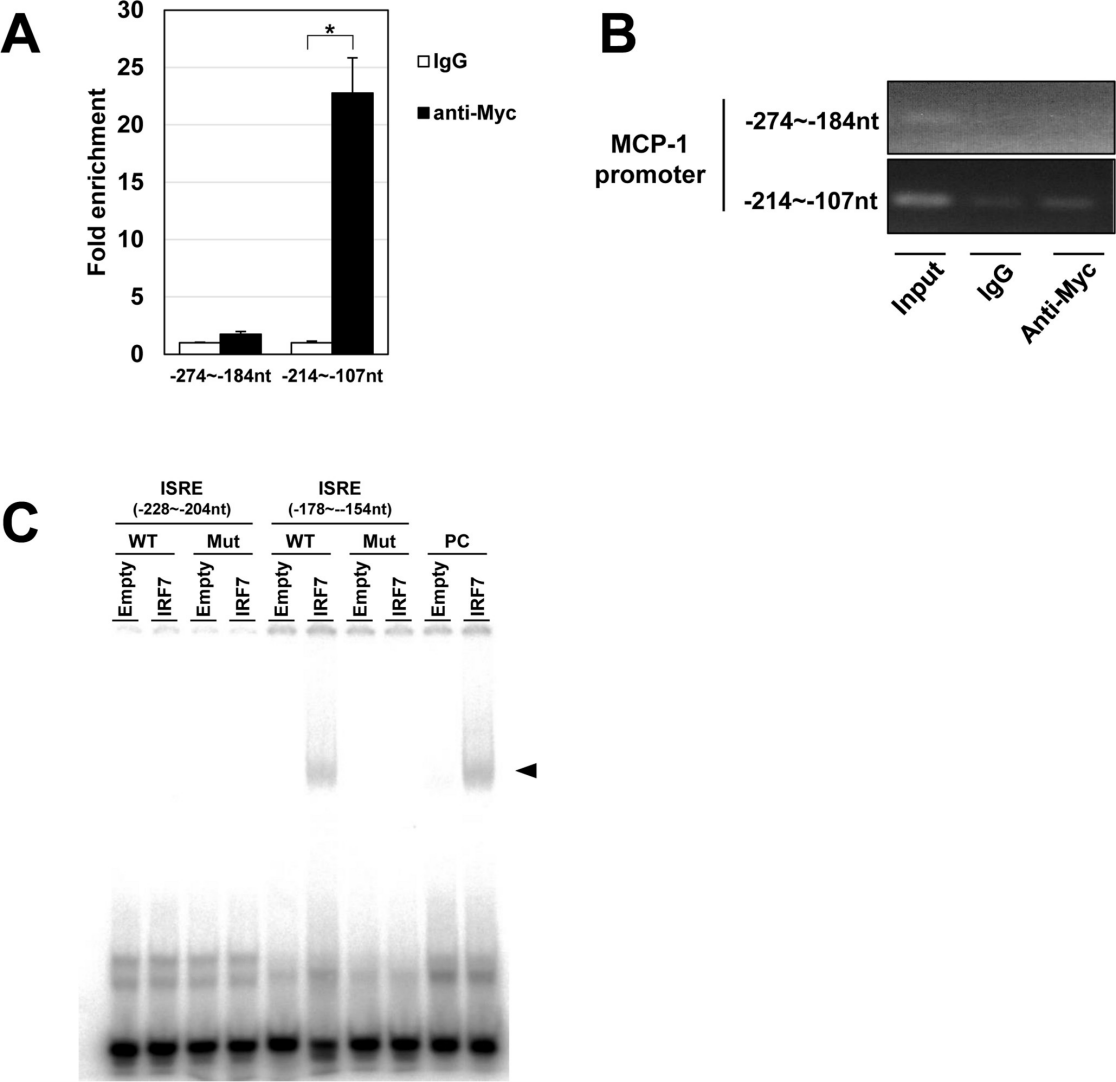
ChIP-PCR analysis of IRF7-myc-C binding to the mouse MCP-1 promoter. A: Quantitative, real-time PCR was performed with the indicated primer pair using an anti-myc antibody immunoprecipitated DNA fragment as template. Normal mouse IgG was used as negative control (n = 4). B: PCR products were separated by agarose gel electrophoresis and detected by ethidium bromide staining. C: DNA and nuclear protein interaction was examined by EMSA. DNA probes for ISRE (−228 to −204 nt) (WT) and its mutant (Mut) and for ISRE (−178 to −154 nt) and its mutant were used. The DNA probes confirmed to bind to IRF7 in the past [14] was used as positive control (PC). The arrowhead indicates protein-DNA complex. *p < 0.05 and **p < 0.01. All values represent mean ± SEM.

Next, we carried out a ChIP-PCR assay to confirm the binding of IRF7 to the more proximal ISRE in the MCP-1 promoter. We used myc in the C-terminal expression vector and transiently transfected it into mature 3T3-L1 adipocytes. We obtained significantly higher amounts of region containing −178 to −154 nt ISRE with anti-myc immuno-precipitation than with IgG control (Fig 5A and 5B). Consistent with this result, EMSA assay showed that IRF7 bound to 25 nt DNA sequence within ISRE (−178 to −154 nt) and mutation within that sequence significantly impaired the binding (Fig 5C). Those results strongly indicate that IRF7 binds to the proximal ISRE and activates MCP-1 transcription.

### MCP-1 expression in IRF7 knockout mice

Our results so far had not confirmed the effects of IRF7 on MCP-1 expression in adipocytes *in vivo*. However, another study demonstrated that IRF7 KO mice showed a markedly lean phenotype, even under high-fat feeding conditions [15], which suggests that it is difficult to evaluate the physiological importance of the transcription factor on hypertrophy-induced MCP-1 production.

**Fig 6:**
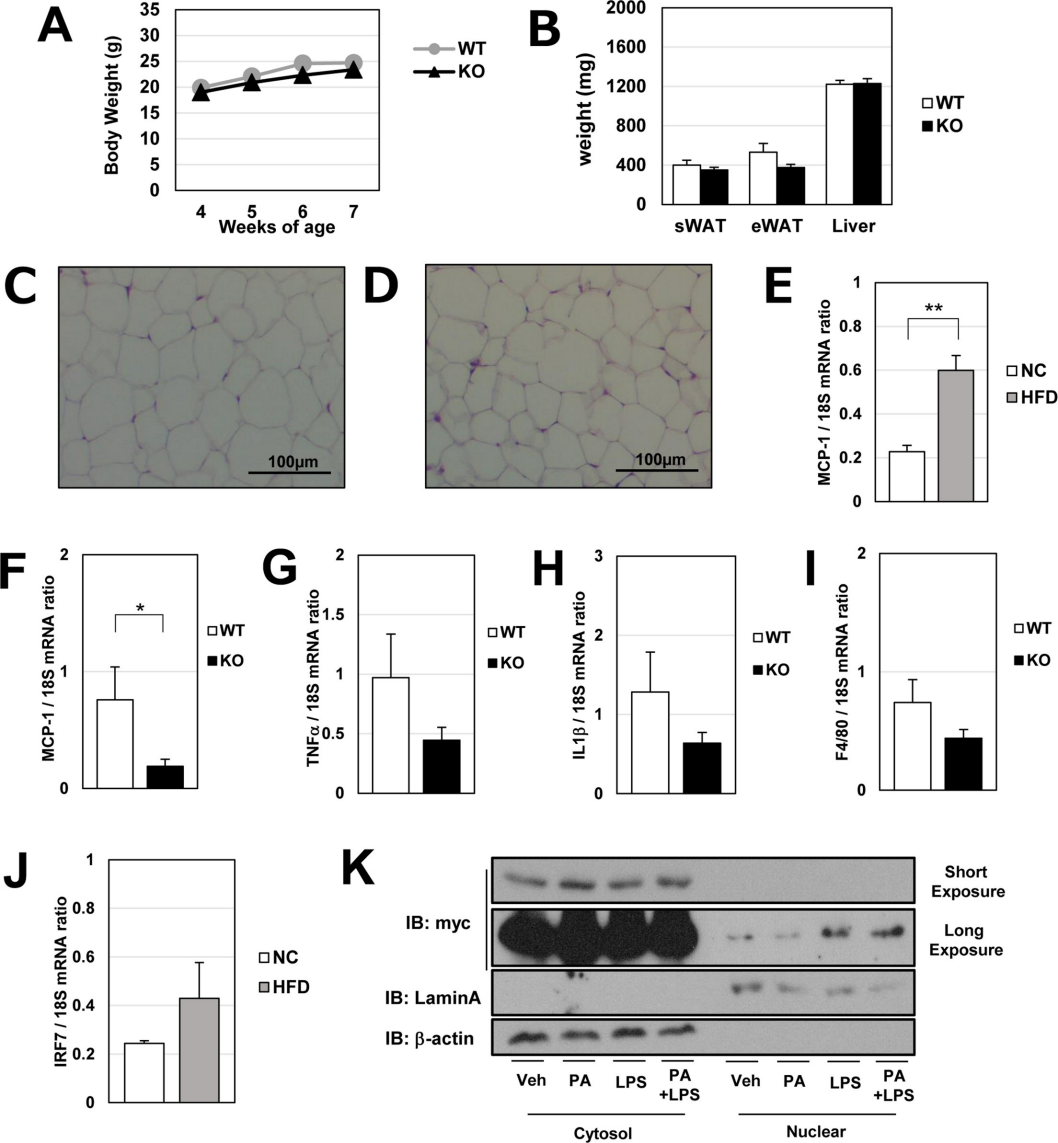
Characteristics of IRF7 knockout mice and effects of short-term high-fat diet (HFD) on IRF7 in adipocytes. A–J: At the age of 4 weeks, IRF7 KO or WT control mice were started on either a HFD or a NC (n = 6–8). After 3 weeks, subcutaneous white adipose tissue (sWAT), eWAT and liver tissue were excised. Body weights were measured during the HFD feeding (A). Excised tissues were weighed (B) and eWAT was subjected to HE staining (C; wild type mice and D: knockout mice). The effect of short-term HFD on MCP-1 mRNA in eWAT was assessed in WT mice (E). The expressions of several inflammatory adipokines (MCP-1, TNF-α, IL1β) and macrophage marker gene (F4/80) were examined by real-time qPCR in WT and KO mice (F–I). IRF7 mRNA levels in eWAT were also measured in WT mice (J). 3T3-L1 adipocytes (at day 5) were transfected with IRF7-myc expression vector. After 24 hours, the cells were treated with 400 μM palmitic acid (PA), 100 ng/ml LPS, or with a combination of both. Cells were lysed and separated into cytosol and nuclear fractions, and subjected to SDS-PAGE and immune blot analysis. *p < 0.05 and **p < 0.01. All values are means ± SEM.

Aside from hypertrophy, MCP-1 is also induced in visceral adipose tissue during the early phase of high-fat feedings [16] when adipocyte hypertrophy has not occurred yet. Therefore, we analyzed mice fed with HFD for only a short-term (3 weeks), before significant differences in body weight, body fat, and the adipocyte cell size (Fig 6A–6D) occurred.

In agreement with another report [8], we confirmed 3 weeks of high-fat feeding increased the MCP-1 mRNA in eWAT of mice (Fig 6E). As shown in Fig 6F, under HFD conditions, the MCP-1 mRNA was lower in KO mouse than in WT controls; on the other hand, we found no differences in other inflammation markers, including TNFα, IL1β, or F4/80 mRNA levels (Fig 6G–6I). Though a short-term HFD did not increase IRF7 transcription (Fig 6J), the mRNA level for MCP-1 was significantly lower in KO mice (Fig 6F) suggesting IRF7 may be activated without changes of its expression level. We found LPS treatment increased IRF7 translocation into the nucleus in 3T3-L1 cells (Fig 6K). Physiologically, IRF7 may be activated through increased endotoxemia in the absence of apparent obesity, and it may be involved in the early induction of MCP-1 in adipose tissues.

## Discussion

MCP-1, also known as C-C motif chemokine ligand 2 (CCL2), is a key player in inflammation. This inflammatory cytokine is produced following infection, binds to the CCR2 receptor on monocytes, and triggers their migration into inflamed lesions. Besides infection, the MCP-1 protein has been involved in other inflammatory conditions and plays a role in the progression of inflammation-dependent diseases, including atherosclerosis [17], arthritis [18], and cancer [19].

During the past few decades, it has been revealed that obesity is associated with chronic systemic inflammation, preceded by activation of the innate immune system within adipose tissues, and this low-grade inflammation links obesity and related-health problems. The adipose tissue is now recognized as an endocrine organ and in the obese mouse model, the expression and secretion of pro-inflammatory cytokines in adipose tissue are elevated [20]. MCP-1 is thought to be an initial trigger leading to further inflammation and to the metabolic disorder associated with obesity. In fact, many studies have shown the importance of MCP-1 and its receptor CCR2 on obesity-related metabolic disorders. Genetic loss of MCP-1 [21] or CCR2 [22] decreases the adipose tissue macrophage content, reduces inflammation, and protects mice from HFD-induced insulin resistance. Pharmaceutical inhibition of CCR2 improves insulin resistance and hepatic steatosis in *db/db* [23] and diet-induced obese mice [24]. Contrary to that, transgenic mice, in which MCP-1 is overexpressed in adipose tissue, exhibit insulin resistance and increased numbers of adipose tissue macrophages [21, 25]. Revealing the regulatory mechanisms of gene expression is important for understanding human diseases. In this study, we found that a transcription factor, IRF7, may regulate MCP-1 expression in fat cells.

IRF7 was originally identified in 1997 [26], and subsequent studies have demonstrated that it was a master regulator of type I interferon induction and of the interferon response gene expression against virus infections. IRF7 is activated upon virus infection, and it stimulates a set of genes involved in host anti-virus defenses. In 2008, Dr. Eguchi and colleagues performed an analysis combining DNase hypersensitivity and computational algorithms to predict transcriptional pathways in adipogenesis [11]. They investigated the important cis-acting element of 27 adipocyte-selective genes in 3T3-L1 cells prior to differentiation and after conversion to mature adipocytes. IRFs were finally identified as transcriptional regulators involved in adipocyte differentiation. Furthermore, they demonstrated that nine IRF members were transcriptionally regulated during adipogenesis and that several IRFs, such as IRF3 and IRF4, negatively affected adipocyte differentiation. Our study further raised the possibility that in addition to adipogenic processes, IRFs could regulate inflammatory changes in adipocytes triggered by nutritional overload. We first sought to identify transcriptional factors associated with the process of adipocyte hypertrophy, rather than adipogenic differentiation, using a combination of DNA microarray and genomic data analysis of binding motif prediction. As a result, we have identified IRFs. Among 9 isoforms of IRFs, we focused on IRF7, which was based on the mRNA expression pattern of IRFs in adipocytes *in vitro* and in adipose tissue from HFD-fed mice. Our *in vitro* experiments show that long-term culture increased IRF7 mRNA expression, which finely parallels that of MCP-1 (Figs 2G and 3B), and loss-of and gain-of-function experiments also support the involvement of IRF7 in MCP-1 expression in adipocytes (Fig 3C–3K). Moreover, MCP-1 mRNA expression in eWAT from IRF7 KO mice was significantly lower than that in WT controls, implying that the transcriptional factor is physiologically important for MCP-1 production.

Jin X et al. previously reported that IRF7 could promote the transcription of pro-inflammatory cytokines, including MCP-1, in gliomas [27]. In the current study, we further discovered that this transcription factor upregulated MCP-1 in adipocytes and might be associated with obesity-induced chronic inflammation in adipose tissue. Notably, we also examined the details of the transcriptional activation of MCP-1 by IRF7. Our promoter assay showed that IRF7 itself induced MCP-1 transcription by binding to its proximal ISRE (–178 to –154 nt). This murine ISRE region was not completely conserved in humans, but interestingly, this region overlapped with the IRF7 motif, which is very similar to the ISRE sequence (S3A Fig) [28, 29]. This suggests that IRF7 may have an important role in MCP-1 expression in humans. Moreover, this transcription factor may amplify MCP-1 production in the presence of inflammatory factors by interacting with other transcription factors. Following LPS stimulation, which activates MyD88 and leads to nuclear translocation of NF-κB, IRF7 overexpressed cells showed the interesting expression pattern of MCP-1 mRNA. As shown in Fig 2B, TAP2 mRNA, a well-known IRF7 target gene [30], was elevated in 3T3-L1 adipocytes by IRF7 over-expression, while it was not affected by LPS treatment. Meanwhile, we observed a significant increase in MCP-1 mRNA after LPS treatment, and the extent of induction by LPS was much larger in IRF7 over-expressed cells than in control cells. A crosstalk between IRF7 and the downstream signals of LPS may exist because the effects of LPS on MCP-1 induction in IRF7 overexpressed cells was not just additive but synergistic.

To exert its transcriptional activity fully, IRF7 needs to form a transcription complex or a virus-activated factor (VAF) by interacting with other proteins, like IRF3, p300, and CBP, and binding to ISRE in the promoter of interferon- and virus- inducible genes. Although the VAF complex may transactivate a variety of genes, it is not sufficient to induce others, such as interferon-β [31]. In virus-infected cells, ATF-2/c-Jun, and NF-κB were recruited to the interferon-β enhancer, forming a highly-coordinated transcriptional complex, enhanceosome, together with IRF3/7, p300, and CBP, resulting in effective interferon-β production [32]. Also, *in vitro* experiments demonstrated that, for full induction of interferon-β, both IRF3/7 and NF-κB need to be activated [32]. Neither type I interferon nor TNF-α alone, which activate either NF-κB or IRFs, were able to induce interferon-β. However, simultaneous stimulation of type I interferon and TNF-α led to a dramatic increase in interferon-β expression [33]. Similar to the regulation on interferon-β, NF-κB might synergistically work with IRF7 for MCP-1 transcription. Interestingly, a sequence similar to the motif for HMG-I/Y which is known to stabilize the binding and assembly of the enhanceosome complex is observed around the distal κB binding site in MCP-1 promoter [34, 35]. Those data indicate transcription factor IRF7 may increase MCP-1 expression in response to inflammatory factors, like LPS, and during basal conditions.

The upstream signaling pathway to induce IRF7 expression during the course of adipocyte hypertrophy is also important. Basically, type I interferon signals or a virus infection are typical IRF7 inducers. In 3T3-L1 adipocytes, IRF7 mRNA was dramatically increased after culturing for 20 days (Fig 1E), but type I interferon was hardly detected at any time point from day 2 to 20, suggesting that type I interferon- and virus infection- independent pathways may be involved. Akira et al. reported that some Toll-Like receptors, like TLR7, 8 and 9, induce antiviral responses via IRF7 activation [36]. Among these three subtypes, our DNA microarray data detected TLR7 and TLR9 in 3T3-L1 adipocytes, and their expression levels increase from day 2 through day 20 (see GSE147858). Of note, TLR9 is reportedly activated by obesity-related DNA-release from adipocytes and is involved in the chronic inflammatory response as well as insulin resistance [37]. Even *in vitro*, adipocytes released double-stranded DNA (dsDNA) into the culture media, and dsDNA acts as a TLR9 ligand to stimulate inflammatory signals [37].

Through analyzing IRF7 knockout mice, we noticed that IRF7 was involved in adipose MCP-1 during early phases of HFD (before adipocyte hypertrophy had appeared in WT animals). Though short-term HFD did not activate IRF7 transcription (Fig 6J), MCP-1 mRNA was certainly upregulated in WT mice, and genetic deletion of IRF7 significantly lowered the chemokine transcription. Those observations imply that IRF7 may be activated with no change in expression level at an early stage of obesity. Obesity-associated systemic inflammation is caused by multiple factors, and a triggering initial event has not been fully elucidated. Burcelin et al. associated intestinal microbiota with early induction of inflammatory chemokines by showing that, shortly after HFD, intestinal permeability increases and bacterial LPS goes into the bloodstream in mice models [38]. This metabolic endotoxemia further triggers acute inflammatory genes, including MCP-1, in adipose tissue [39]. IRF7 transcriptional activity is also regulated by its intracellular localization. We found that LPS treatment increased IRF7 translocation to the nucleus in 3T3-L1 adipocytes (Fig 6K). We were unable to estimate the physiological importance of IRF7 in hypertrophy-mediated MCP-1 induction in fat cells, because of the strong resistance to obesity in knockout mice. However, we found IRF7 could also be activated independently of the lipid overload in adipocytes and that it could be associated with MCP-1 production possibly through increased endotoxemia in obese individuals.

## Conclusions

We revealed the transcription factor IRF7 was involved in the pathogenesis of obesity, presumably by regulating the pro-inflammatory adipokine MCP-1. The functional annotation analysis was performed on the highly expressed genes that are shared in IRF7 over-expressed and long term cultured adipocytes and we found that there was a functional enrichment in “immunity”, “innate immunity”,” antiviral defense”,”MHC-I”, and so on (S2 Table). This result may imply IRF7 dose not only affect the MCP-1 transcription but also might be involved in more extensive immune system in fat cells.

Moreover, Li H et al. reported that IRF7 knockout mice were protected against high-fat induced obesity and its related metabolic abnormalities [15], suggesting that, in addition to transcriptionally regulating MCP-1, IRF7 may be involved in energy homeostasis by unknown mechanisms. Blockage of IRF7 or its upstream pathways could become a therapeutic target.

## Supporting information

S1 Table List of genes that were upregulated in 3T3-L1 adipocytes both by enforced expression of IRF7 and by long-term cell culture. (XLSX)

S2 Table The result of functional annotation analysis on the genes that were commonly upregulated by IRF7 over expression and by long term cell culture. (XLSX)

S1 Fig Effects of long-term cell culture on adipocyte biology. A-D: 3T3-L1 cells 2 (A), 8 (B) and 20 days (C) after the induction of adipogenesis were stained with oil-red. Photographs were obtained by phase-contrast microscopy. Accumulated dye was extracted with isopropanol, and 492nm absorbance was measured (D)(n = 5). E-F: 3T3-L1 adipocytes were fixed with osmium tetroxide and suspended in Isoton^TM^ II Dilutant (Beckman, CA, USA). Cell size and its distributions (E), mean diameter (F)(n = 4–6) were measured by a Coulter Multisizer3 (Beckman, CA, USA). G-J: 3T3-L1 adipocytes at day 2, 8, and 20 were harvested and isolated mRNA were used for gene expression analysis. *p < 0.05 and **p < 0.01. All values are means ± SEM.
(TIF)

S2 Fig The effects of forced expression of IRF7 on insulin receptor signaling and 2-deoxyglucose transport. A: control- (retro-Empty)(A) or IRF7 overexpressed- (retro-empty)(B) mature adipocytes at day 7 were fixed with 3.7% formaldehyde and stained with Oil-Red O. C: Accumulated dye was extracted with isopropanol, and 492nm absorbance was measured (n = 6). D: After 5 hours serum starvation, IRF7 overexpressed- (retro-IRF7) or control (retro-empty) adipocytes were stimulated with 10^−9^ or 10^−7^ M insulin for 5 min. Cells were harvested with lysis buffer containing β-glycerophosphate and sodium orthovanadate, and subjected to immunoblot of phosphor-ERK and phosphor-Akt. E: After 5 hours serum starvation, retro-Empty or retro-IRF7 adipocytes were stimulated with 10^−9^ or 10^−7^ M insulin, followed by 1 mM 2-deoxyglucose. Transported 2-DG amount was normalized to the total cellular protein (n = 6). *p < 0.05 and **p < 0.01. All values are means ± SEM.
(TIF)

S3 Fig Alignment of MCP-1 promoter sequence of mouse and human. A: the 5’-flanking regions of human (–227 to –133 nt) and mouse (–238 to –143 nt) MCP-1 gene were compared. Putative transcription factor binding sites were indicated by boxing.
(TIF)

S1 Raw images (PDF)
