# Supplementary figures and images for: Interferon regulatory factor 7 mediates obesity-associated MCP-1 transcription
Source: PLoS One. 2020 May 21;15(5):e0233390. doi: 10.1371/journal.pone.0233390 (PMC7241760; doi:10.1371/journal.pone.0233390)

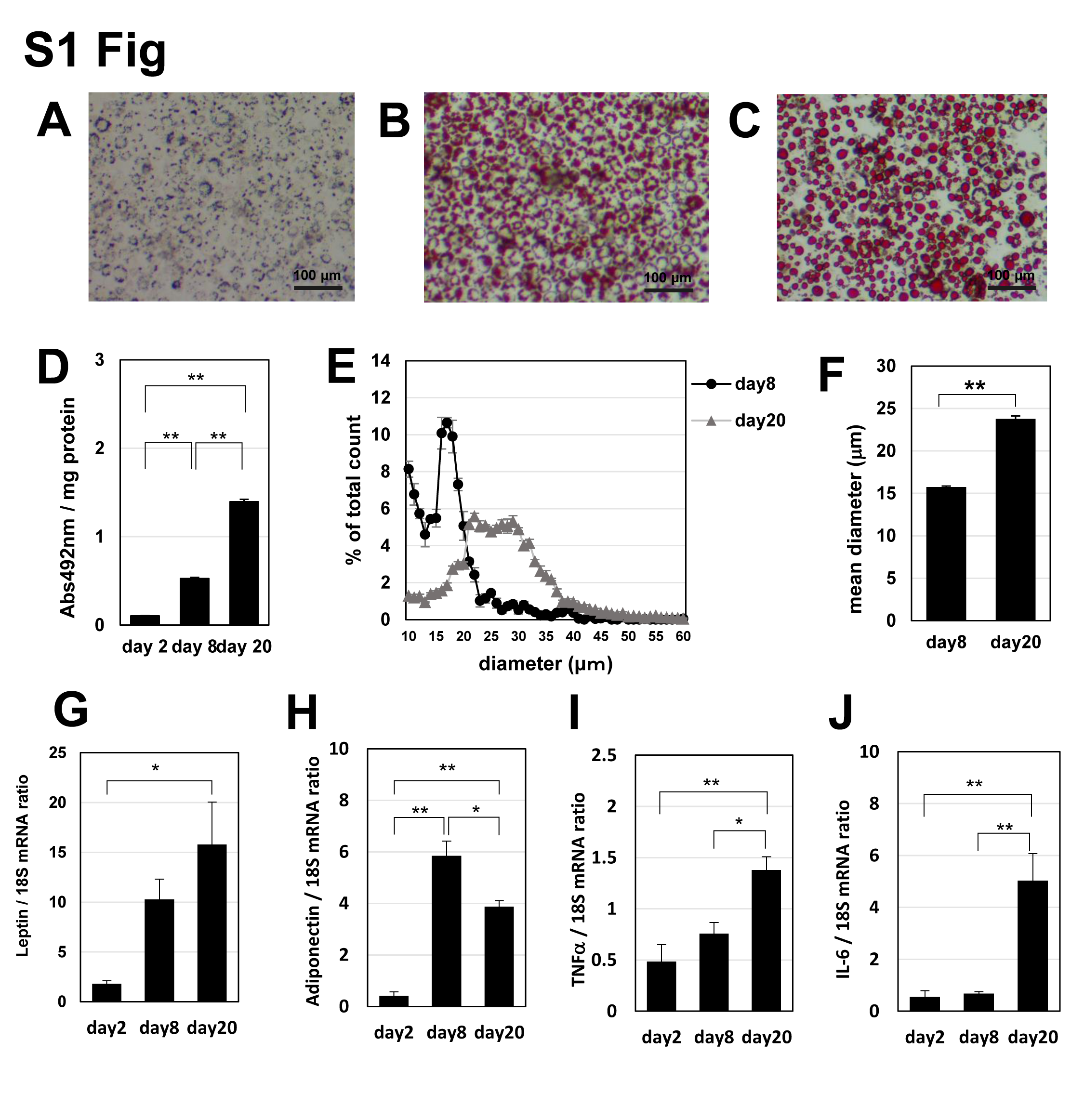

Supplement: S1 Fig — A-D: 3T3-L1 cells 2 (A), 8 (B) and 20 days (C) after the induction of adipogenesis were stained with oil-red. Photographs were obtained by phase-contrast microscopy. Accumulated dye was extracted with isopropanol, and 492nm absorbance was measured (D)(n = 5). E-F: 3T3-L1 adipocytes were fixed with osmium tetroxide and suspended in IsotonTM II Dilutant (Beckman, CA, USA). Cell size and its distributions (E), mean diameter (F)(n = 4–6) were measured by a Coulter Multisizer3 (Beckman, CA, USA). G-J: 3T3-L1 adipocytes at day 2, 8, and 20 were harvested and isolated mRNA were used for gene expression analysis. *p < 0.05 and **p < 0.01. All values are means ± SEM. (TIF) [file pone.0233390.s003.tif]

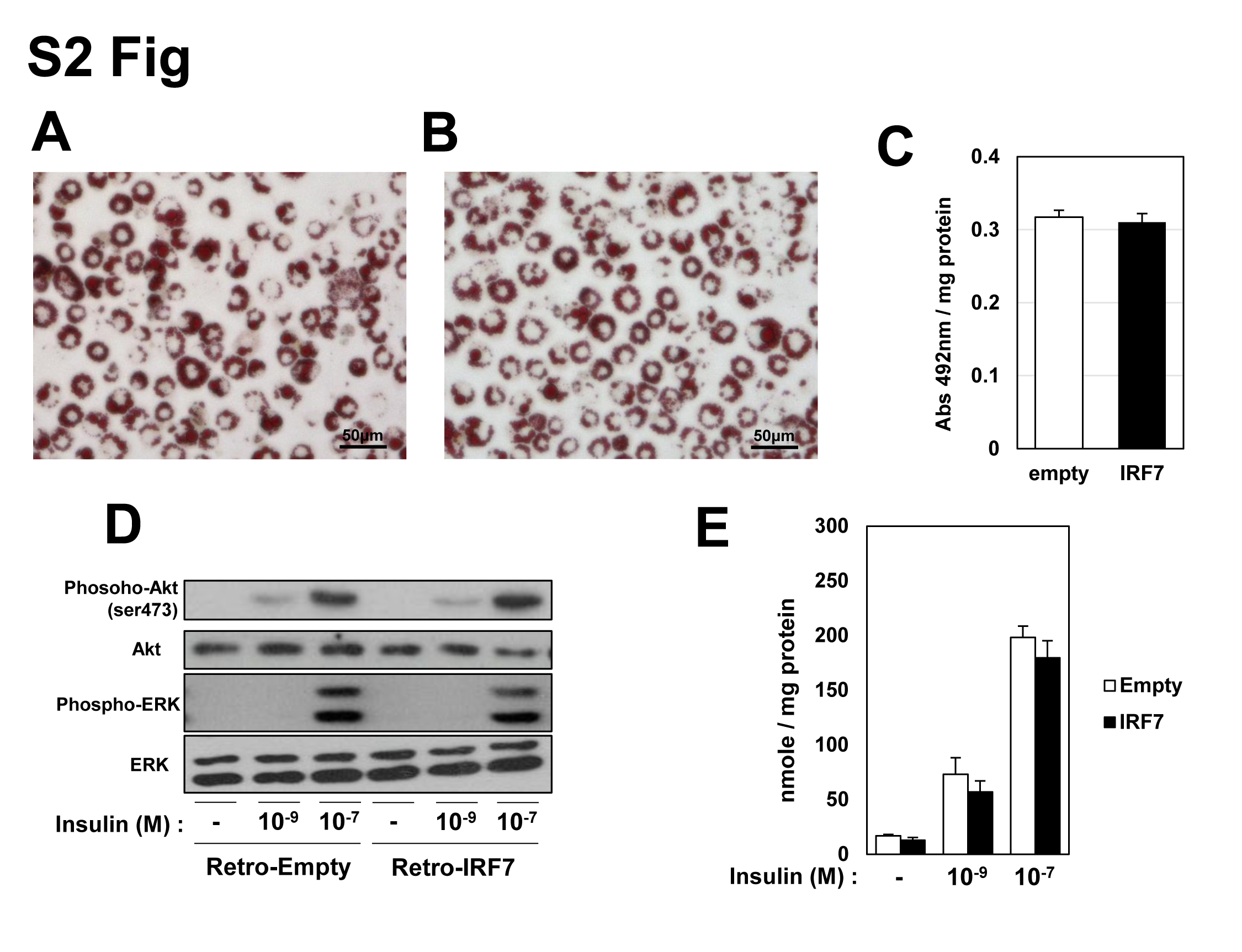

Supplement: S2 Fig — A: control- (retro-Empty)(A) or IRF7 overexpressed- (retro-empty)(B) mature adipocytes at day 7 were fixed with 3.7% formaldehyde and stained with Oil-Red O. C: Accumulated dye was extracted with isopropanol, and 492nm absorbance was measured (n = 6). D: After 5 hours serum starvation, IRF7 overexpressed- (retro-IRF7) or control (retro-empty) adipocytes were stimulated with 10−9 or 10−7 M insulin for 5 min. Cells were harvested with lysis buffer containing β-glycerophosphate and sodium orthovanadate, and subjected to immunoblot of phosphor-ERK and phosphor-Akt. E: After 5 hours serum starvation, retro-Empty or retro-IRF7 adipocytes were stimulated with 10−9 or 10−7 M insulin, followed by 1 mM 2-deoxyglucose. Transported 2-DG amount was normalized to the total cellular protein (n = 6). *p < 0.05 and **p < 0.01. All values are means ± SEM. (TIF) [file pone.0233390.s004.tif]

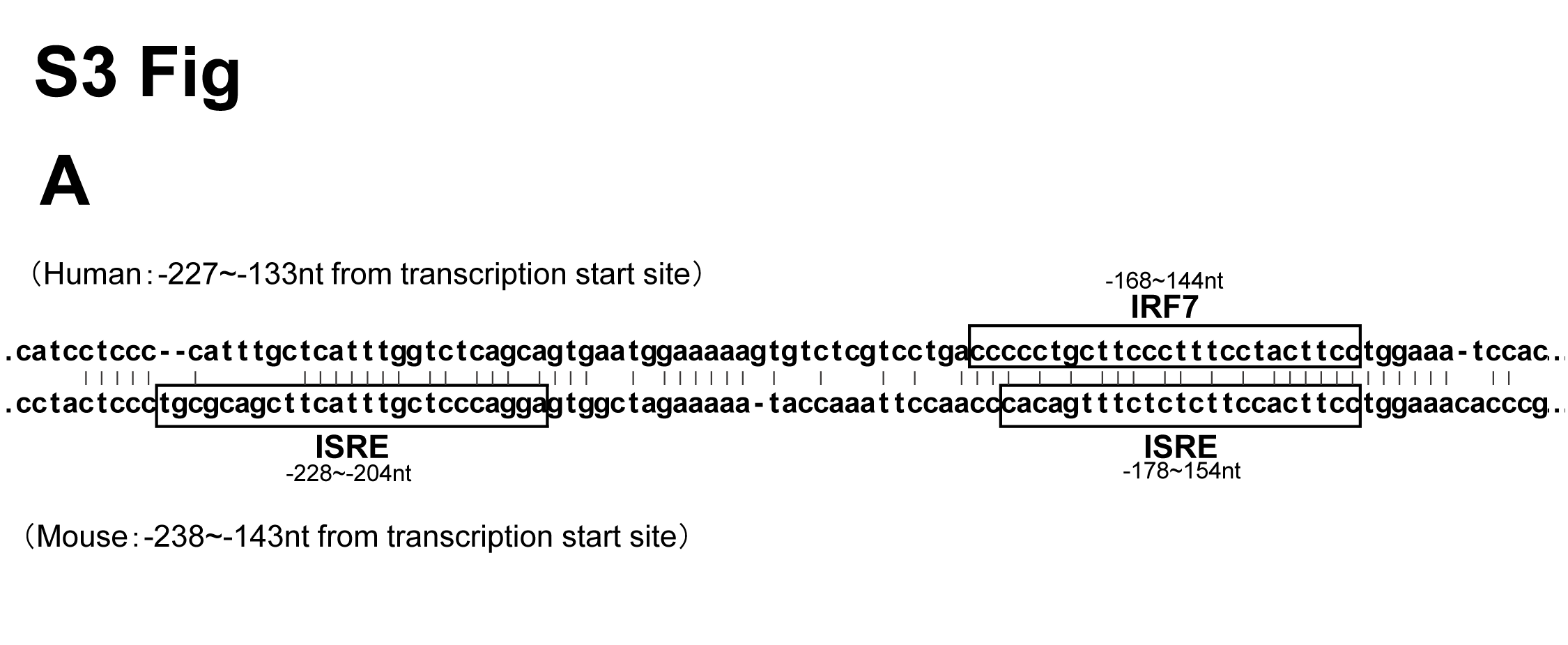

Supplement: S3 Fig — A: the 5’-flanking regions of human (–227 to –133 nt) and mouse (–238 to –143 nt) MCP-1 gene were compared. Putative transcription factor binding sites were indicated by boxing. (TIF) [file pone.0233390.s005.tif]
