# Supplementary material for: Interferon regulatory factor 7 mediates obesity-associated MCP-1 transcription
Source: PLoS One. 2020 May 21;15(5):e0233390. doi: 10.1371/journal.pone.0233390 (PMC7241760; doi:10.1371/journal.pone.0233390)
Supplement: S1 Raw images — (PDF) [file pone.0233390.s006.pdf]

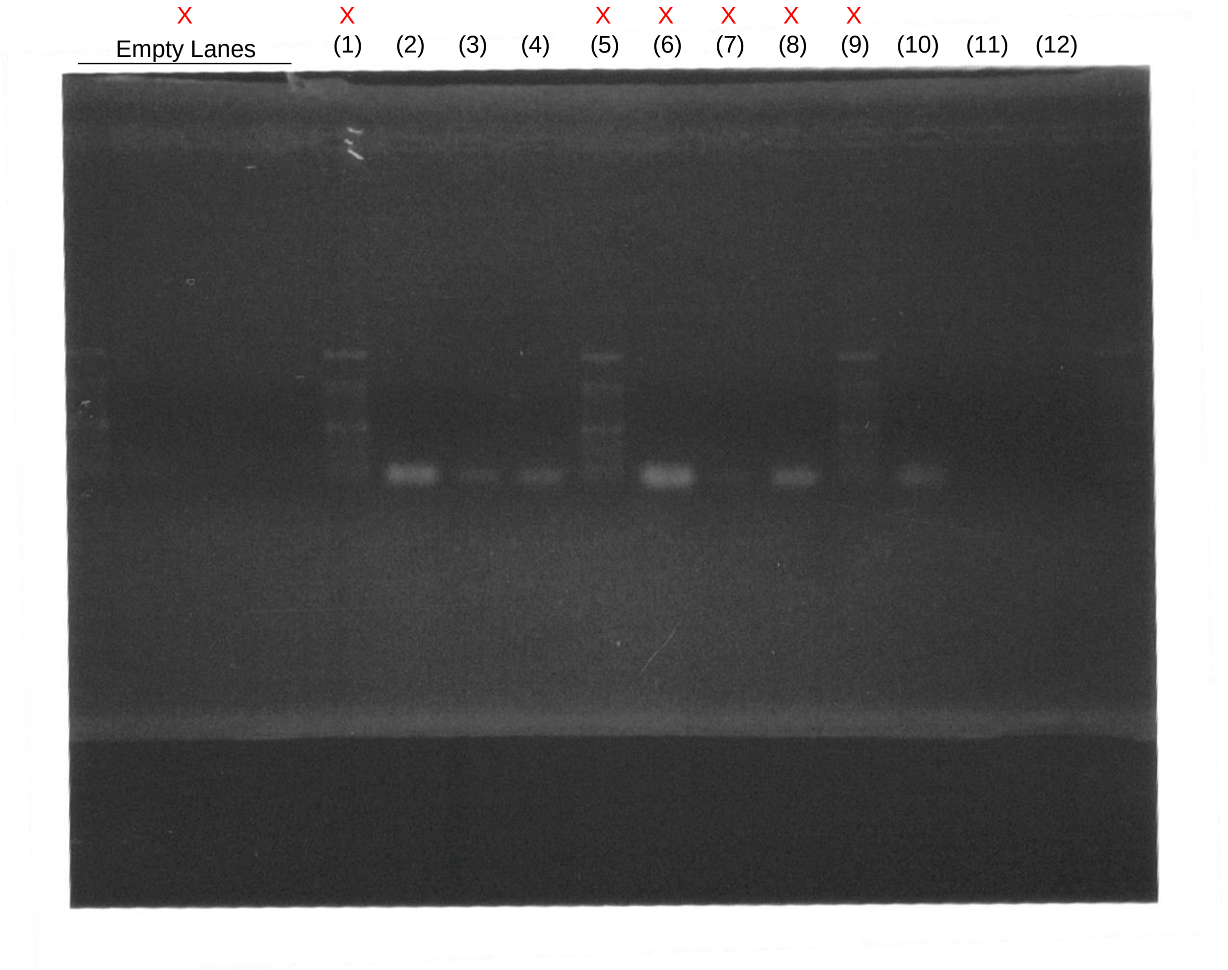

S1_raw_Fig 5B: Detection of ChIP-PCR product by agarose gel electrophoresis and ethidium bromide staining. Lane 1, 5 and 9: 100bp DNA Ladder (TaKaRa, Tokyo, Japan). Lane 2-4 and 6-8: PCR product of primer pairs (-214~-107nt from transcription start site of mouse MCP-1). PCR templates were Input gDNA(Lane 2 and 6), and normal mouse IgG (Lane 3 and 7) and anti-myc anti-body (Lane 4 and 8) immunoprecipitated DNA fragment. PCR products in Lane 2-4 and Lane 6-8 were obtained from independent experiments with different sample preparations. Lane 10-12: PCR product of primer pairs -274~-184nt from transcription start site of mouse MCP-1). PCR templates were Input gDNA(Lane 10), and normal mouse IgG (Lane 11) and anti-myc anti-body (Lane 12) immunoprecipitated DNA fragment. Agarose gel stained with ethidium bromide was visualized using PrintGraph (Atto, Tokyo, Japan). Red crosses indicate wells not included in the final figures. Lane 2-4 and 10-12 were used in Fig 5B.

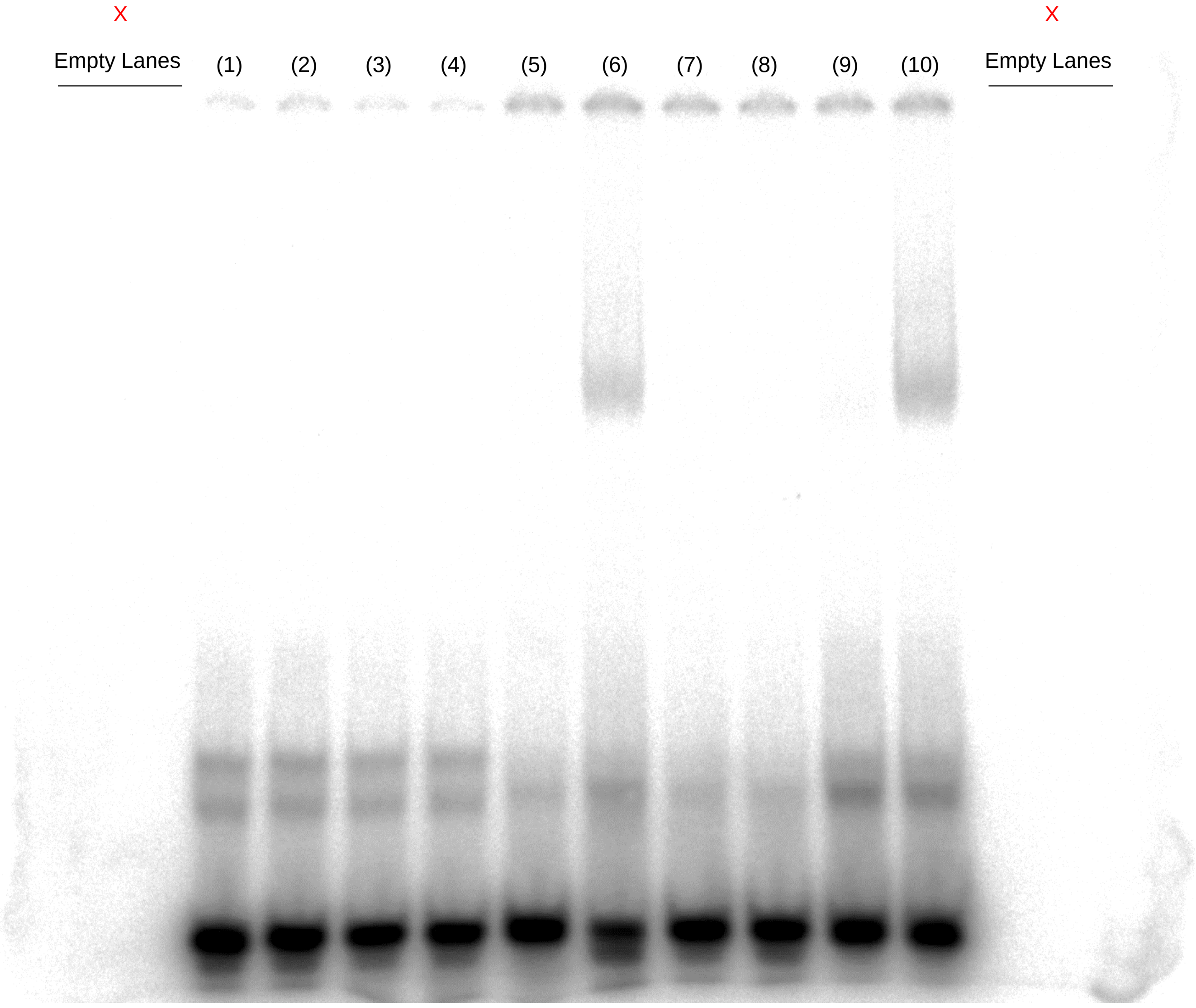

S1_raw_Fig 5C: Electrophoresis Mobility Shift Assay. Lane 1-4: Nuclear protein was extracted from HEK 293 cells transfected with Empty-pcDNA3.1(+) (Lane 1 and 3) or IRF7-pcDNA3.1(+) (Lane 2 and 4).Extracts were incubated with ^{32}P labeled double strand DNA fragment corresponding to ISRE (-228~-204nt from transcription start site of mouse MCP-1) (Lane 1 and 2) and its mutant (Lane 3 and 4). Lane 5-8: Nuclear protein was extracted from HEK 293 cells transfected with Empty-pcDNA3.1(+) (Lane 5 and 7) or IRF7-pcDNA3.1(+) (Lane 6 and 8).Extracts were incubated with ^{32}P labeled double strand DNA fragment corresponding to ISRE (-228~-204nt from transcription start site of mouse MCP-1) (Lane 5 and 6) and its mutant (Lane 7 and 8). Lane 9 and 10: Nuclear protein was extracted from HEK 293 cells transfected with Empty-pcDNA3.1(+) (Lane 9) or IRF7-pcDNA3.1(+) (Lane 10).Protein extracts were incubated with ^{32}P labeled double strand DNA fragment, which was confirmed to bind to IRF7 protein in the past study (J Biol Chem. 275: 34320-34327, 2000.). Lane 9 and 10 were considered as positive control. Samples were separated in 5% polyacrylamide gel and detected using Fluorescent Imaging Analyzer FLA-9000 equipped with Multi-Gauge Version 3.0 software. Red crosses indicate wells not included in final figures. All the lanes were used in Fig 5C.

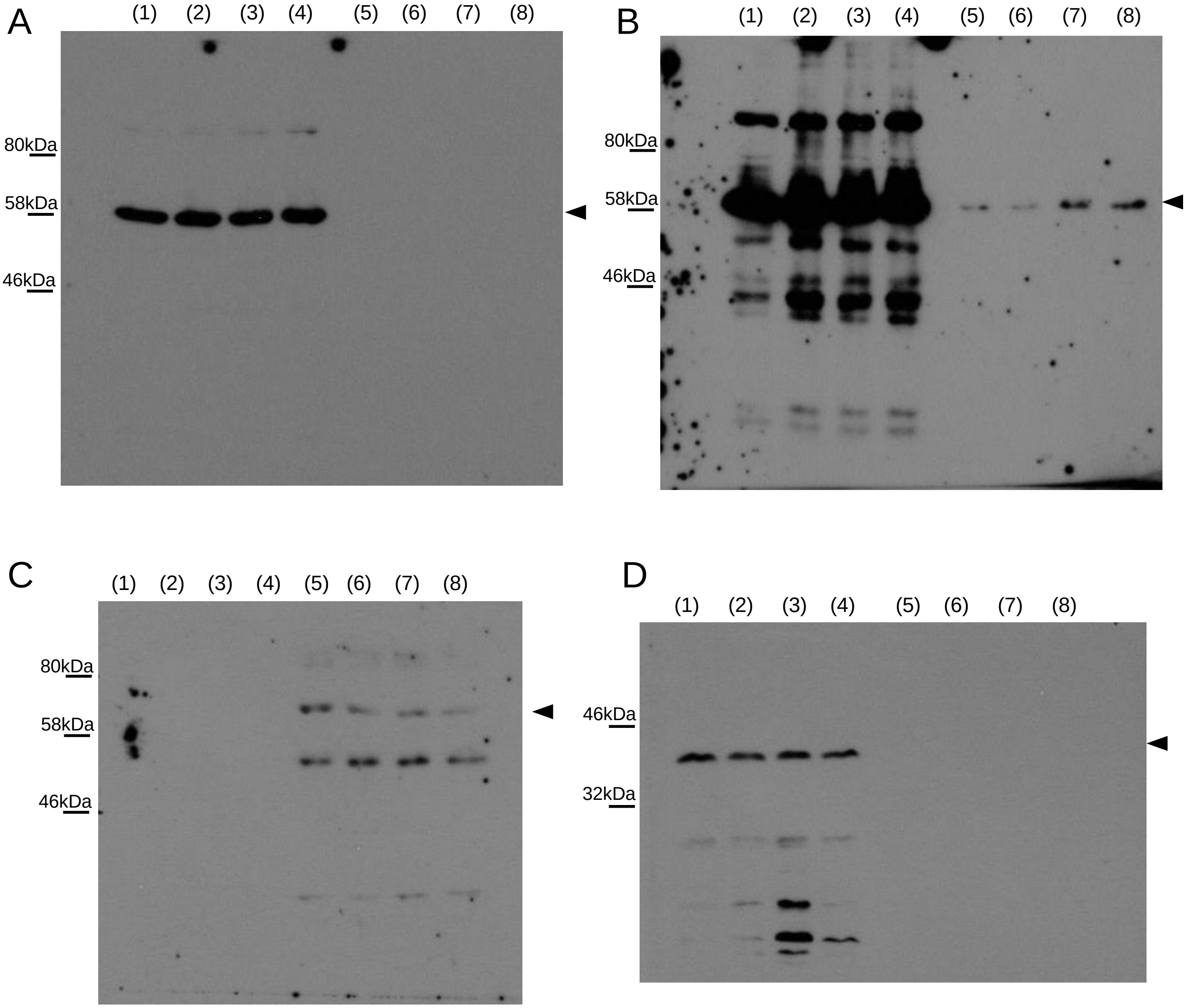

S1_raw_Fig 6K: Western Blotting Images. Lane 1-8: 3T3-L1 adipocytes were transfected with IRF7-myc in pcDNA3.1(+) at day 5. 24 hours after that, vehicle (Lane 1 and 4), 400 μM palmitate (Lane 2 and 6), 100 ng/mg LPS (Lane 3 and 7) or a combination of both (Lane 4 and 8) was added to culture medium and cells were incubated for 24 hours more. Cells were lysed and separated into cytosol (Lane 1-4) and nuclear fraction (Lane 5-8). Equal amount of protein samples were loaded on 8-12 % of polyacrylamide gel and transferred to PVDF membrane. Then, membrane was incubated with anti-myc (expected molecular weight: 60-65 kDa)(Panel A and B), anti-Lamine A (expected molecular weight: 70-80 kDa)(Panel C) and anti-β-actin (expected molecular weight: 40 kDa)(Panel D) antibody. Signals were detected using a Clarity western ECL substrate (BioRad, Hercules, CA) and X-ray film (Carestream, NY, USA). The image of Panel B was captured by long-exposure. The arrowhead indicate the expected position of each protein. All the lanes were used in Fig 6K.

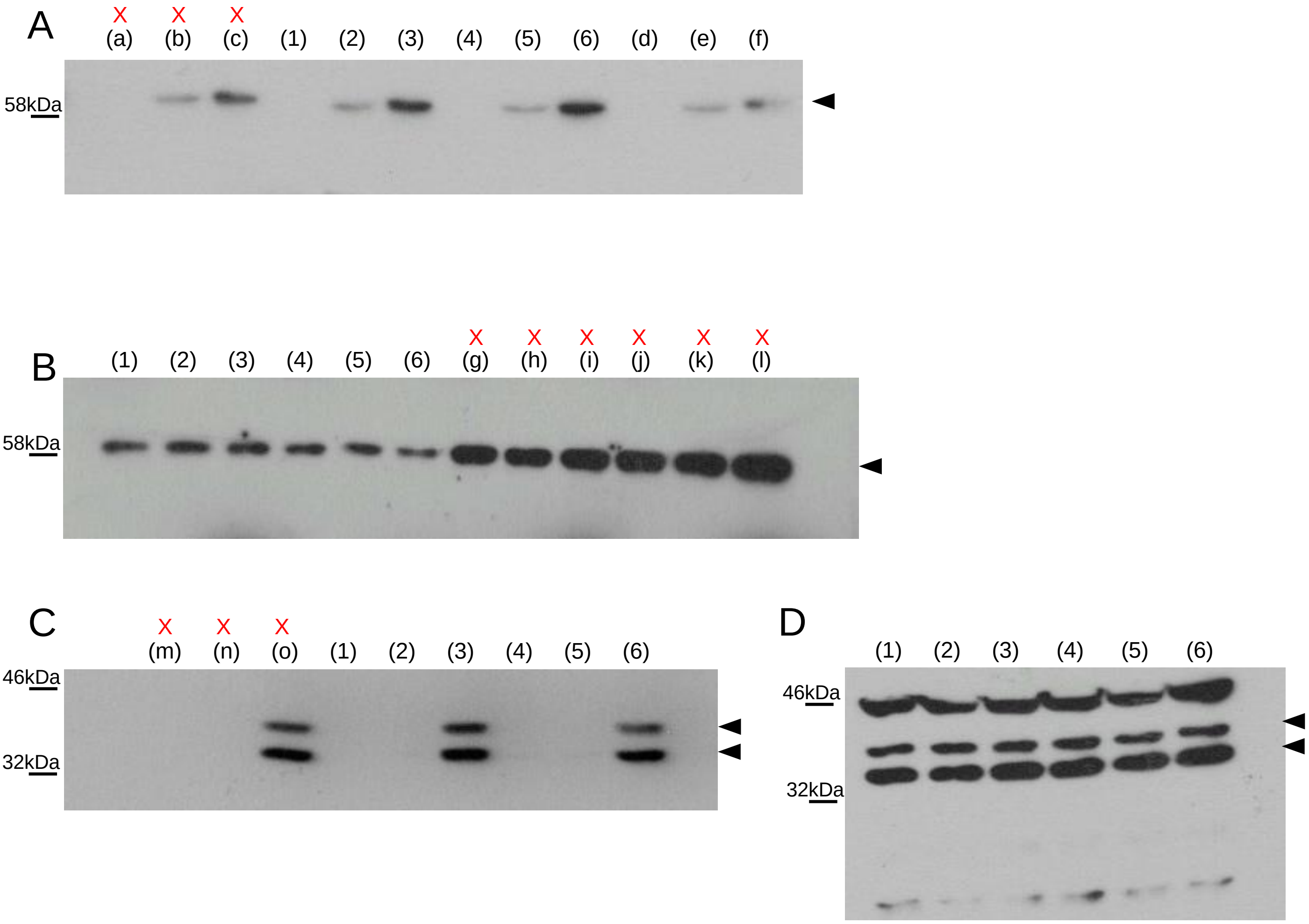

S1_raw_S1 Fig D: Western Blotting Images. Lane 1-6: Whole cell lysate isolated from 3T3-L1 adipocytes treated with various concentrations of insulin. 3T3-L1 adipocytes were differentiated from precursor cells infected with Emptry- (Lane 1-3) or IRF7- (Lane 4-6) retrovirus. On day 7 after the induction of adipogenic differentiation, 3T3-L1 adipocytes were stimulated with 10^{-9} (Lane 2 and 5) or 10^{-7} M (Lane 3 and 6) insulin for 5 minutes. 10 mM HCl was added to culture medium as vehicle control (Lane 1 and 4). Then, cells were lysed with RIPA buffer and isolated cell lyasates were loaded on 8-12 % of polyacrylamide gel and transferred to PVDF membrane. The membrane was incubated with anti phosho-Akt (expected molecular weight: 60 kDa)(Panel A), anti-Akt (expected molecular weight: 60 kDa)(Panel B), anti phosphor-ERK (expected molecular weight: 42/44 kDa)(Panel C) and anti-ERK (expected molecular weight: 42/44 kDa)(Panel D) antibody. Signals were detected using a Clarity western ECL substrate (BioRad, Hercules, CA) and X-ray film (Carestream, NY, USA). The arrowhead indicate the expected position of each protein. Lane (a)-(o): Protein samples un-related to this study. Red crosses indicate wells not included in final figures. Lane 1-6 were used in S2 Fig D.
